# Loss of β4-spectrin impairs Na_v_ channel clustering at the heminode and temporal fidelity of presynaptic spikes in developing auditory brain

**DOI:** 10.1038/s41598-022-09856-9

**Published:** 2022-04-07

**Authors:** Kaila Nip, Sean Kashiwagura, Jun Hee Kim

**Affiliations:** The Department of Cellular and Integrative Physiology, University of Texas Health Science Center, San Antonio, TX 78229 USA

**Keywords:** Auditory system, Cellular neuroscience, Ion channels in the nervous system

## Abstract

Beta-4 (β4)-spectrin, encoded by the gene *Sptbn4*, is a cytoskeleton protein found at nodes and the axon initial segments (AIS). *Sptbn4* mutations are associated with myopathy, neuropathy, and auditory deficits in humans. Related to auditory dysfunction, however, the expression and roles of β4-spectrin at axon segments along the myelinated axon in the developing auditory brain are not well explored. We found during postnatal development, β4-spectrin is critical for voltage-gated sodium channel (Na_v_) clustering at the heminode along the nerve terminal, but not for the formation of nodal and AIS structures in the auditory brainstem. Presynaptic terminal recordings in *Sptbn4*^*geo*^ mice, β4-spectrin null mice, showed an elevated threshold of action potential and increased failures during action potential train at high-frequency. *Sptbn4*^*geo*^ mice exhibited a slower central conduction and showed no startle responses, but had normal cochlear function. Taken together, the lack of β4-spectrin impairs Na_v_ clustering at the heminode along the nerve terminal and the temporal fidelity and reliability of presynaptic spikes, leading to central auditory processing deficits during postnatal development.

## Introduction

Spontaneous or targeted mutations in *Sptbn4*, a gene encoding β4-spectrin, cause severe neurological dysfunction that includes motor dysfunction, myopathy, and auditory neuropathy in humans^1–3^. A *quivering* (*qv*) mouse with mutations in *Sptbn4* is considered an animal model of central deafness. In *qv*^*4j*^ mutant mice, the accuracy of action potential generation in the cochlear nucleus was severely reduced and phase-locked responses to sinusoidal amplitude modulation were greatly diminished^4^. However, the cellular mechanisms of how loss of *Sptbn4* causes impairments in auditory transmission and processing along the central auditory pathway are not well understood.

The calyx of Held is a giant axon terminal on neurons in the medial nucleus of the trapezoid body (MNTB), one of main nuclei in the auditory brainstem. The calyx terminal has prominent physiological features related to fast and high-fidelity processing of temporal information in the auditory nervous system. In particular, clustering of voltage-gated sodium (Na_v_) channels at the heminode, an unmyelinated distal segment in the calyx axon, is essential for tuning propagated action potentials and neurotransmission in the auditory brainstem^5,6^. The heminode of the calyx axon undergoes structural refinement and is influenced by auditory experience during the postnatal development^6^. However, the molecular mechanisms underlying Na_v_ channel clustering at the nerve terminal remains unexplored. We tested the hypothesis that β4-spectrin is important for Na_v_ channel clustering at the calyx of Held terminal to maintain the temporal fidelity of presynaptic spikes in the mammalian auditory nervous system.

Previous studies demonstrated that spectrins such as β4-spectrin are critical for the assembly and maintenance of the AIS and the nodes of Ranvier^7–9^. In cerebellar and hippocampal neurons from β4-spectrin-null mice, Na_v_ channels are not normally clustered at nodes and the AIS, indicating impaired action potential generation and propagation^7,8,10^. In addition, β4-spectrin is important for maintaining the stability of nodal and AIS structure including Na_v_ channel clustering rather than formation^11^. Notably, β1-spectrin with ankyrin-R partially compensates for loss of β4-spectrin to maintain Na_v_ channels at nodes, but not the AIS, suggesting that different β4-spectrin subtypes contribute to domain-specific mechanisms for Na_v_ channel clustering^12–14^. Although roles of β4-spectrin at the node and AIS in the CNS are well determined, it remains unexplored how β4-spectrin expression impacts Na_v_ channel clustering at the last heminode, which is an important axon segment for the tuning of action potentials at presynaptic terminals. Here we demonstrated the presence of β4-spectrin at the heminode and its roles in Na_v_ channel clustering and presynaptic spiking with fast and high temporal fidelity at the nerve terminal during postnatal development. Furthermore, we addressed how loss of *Sptbn4* impacts the auditory transmission and processing along the central auditory pathway.

## Results

### β4-spectrin expression and Na_v_ channel clustering at the heminode occur at a later time point than at the AIS and nodes in the auditory brainstem during postnatal development

**Figure 1:**
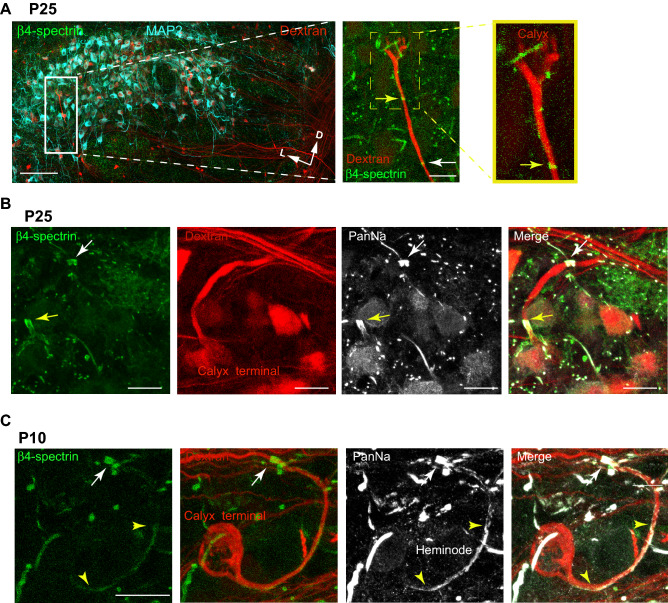
β4-spectrin is expressed in the AIS, nodes of Ranvier and the heminode in the MNTB from mouse auditory brainstem. (**A**) Representative confocal images of the MNTB from WT mouse (at P25). Axon projections were labelled with tetramehtylrhodamine dextran (Dextran, red). MNTB principal neurons and β4-spectrin were labelled with anti-MAP2 (cyan) and anti-β4-spectrin antibody (green). Arrows indicate a lateral (M) or dorsal (D) direction from the midline of the brainstem. Scale bar, 100 µm. The magnified image of the calyx of Held (white box), heminode near the nerve terminal (yellow box), and node of Ranvier. β4-spectrin was expressed at the heminode (yellow arrow) and node (white arrow) along the calyx axon terminal (red). Scale bar, 20 µm. (**B–C**) Confocal images of the calyx of Held terminal labelled with dextran (red), β4-spectrin (green), and Na_v_ channels with anti-PanNa antibody (gray) at P25 (**B**) and P10 (**C**). Yellow arrows indicate β4-spectrin at the heminode and white arrows indicate β4-spectrin at the node of Ranvier. Scale bar, 20 µm.

The myelinated axon of globular bushy cell in the cochlear nucleus, referred as the calyx axon, crosses over the midline and projects contralaterally to the MNTB. We examined β4-spectrin expression in axonal domains (e.g. AIS, nodes, and the heminode) in the MNTB from mouse auditory brainstem (P25). MNTB neurons were immunostained with MAP2 and the calyx axons were anterograde-traced with tetramethylrhodamine dextran staining^15^, Fig. 1A). β4-spectrin was distinctly expressed in the AIS of MAP2 positive neurons, nodes of Ranvier, and the heminode next to the calyx terminal, but not in the presynaptic terminal in the MNTB (Supplemental Fig. 1). β4-spectrin expression has been linked to Na_v_ channel expression at the AIS and nodes in the sciatic nerve, spinal cord, hippocampus, cerebellum and cerebral cortex^8,11^. Similarly, in the auditory brainstem, β4-spectrin was well co-localized with Na_v_ channels at the heminode near the calyx terminal (at P25, Fig. 1B).

β4-spectrin expression was also associated with ankyrin-G (AnkG), which is required for β4-spectrin localization at nodes^16^. In the MNTB, β4-spectrin was well co-localized with AnkG at the AIS and nodes as early as the first week of postnatal age (P9, Supplementary Fig. 1). Notably, in immature calyx terminals at P8-10 when AnkG immunoreactivity was not distinctly detected at the heminode, Na_v_ channels at the heminode were sparsely expressed, not forming the Na_v_ channel cluster ^6^. We examined whether β4-spectrin expression at the heminode is associated with Na_v_ channel clustering in immature calyx axon during postnatal development (at P10). A clustering region with relatively high-intensity staining for Na_v_ channels was detected at nodes (normalized fluorescence intensity > 0.60) as described in a previous study^6^. In young mice brainstem (at P9) the clusters of AnkG, β4-spectrin, and Na_v_ channels were distinctly formed at nodes. In contrast, the expression of β4-spectrin and AnkG were very weak and dispersed along the heminode in the immature calyx. Accordingly, Na_v_ channels were not formed as a cluster at the heminode yet (Fig. 1C, Supplementary Fig. 1). Thus, during postnatal development, β4-spectrin expression and clustering at the heminode along the nerve terminal occurs at a later timepoint than at the AIS or nodes in the auditory brainstem.

### β4-spectrin is important for Na_v_ channel clustering at the nerve terminal during postnatal development

**Figure 2:**
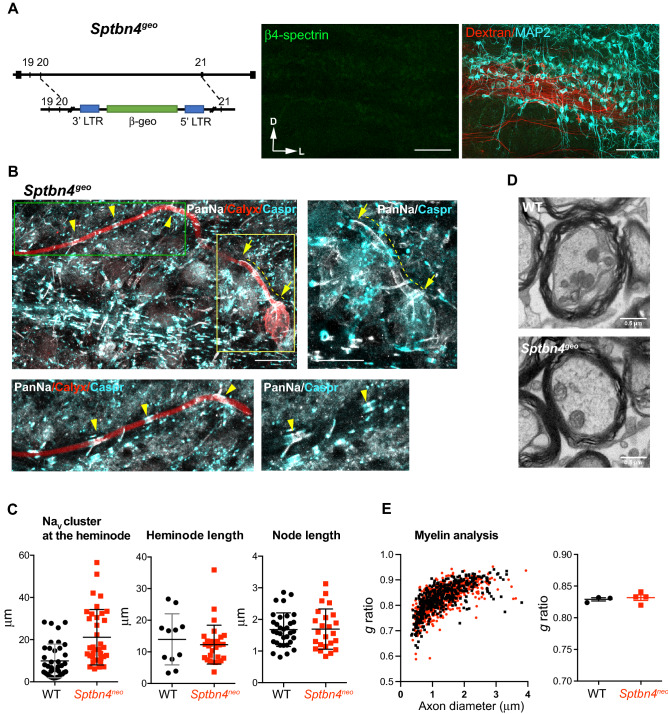
Loss of β4-spectrin impairs Na_v_ clustering at the heminode in myelinated axons. (**A**) (Left) Schematic of the Sptbn4 locus in *Sptbn4*^*geo*^ mice, the gene trap insertion of Rosaßgeo is between exons 20 and 21. (Right) Image of the MNTB from *Sptbn4*^*geo*^ mouse (at P25) with axon projections labelled with Dextran (red). MNTB principal neurons were labelled with anti-MAP2 (cyan) and anti-ß4-spectrin antibody (green). Scale bar, 100 µm. (**B**) The calyx of Held terminal of *Sptbn4*^*geo*^ mouse (at P17) was dye-filled during whole-cell recording, then post-immunostaining with anti-NaPan antibody (gray) and anti-Caspr (cyan). (Top right) The crop image of box area (yellow). Arrows and dotted lines indicate the edges and length of Na channel expression in the heminode, respectively. (Bottom left) Magnified image of the calyx (green box in Top left). Yellow arrowheads indicate Na_v_ channels at nodes (gray), which flanked with Caspr at paranodes (cyan). Scale bar, 20 µm. (**C**) Summary of node length (µm), Na_v_ cluster size, (µm) and heminode length (µm). Values are means ± S.D., ***p < 0.001, Mann–Whitney U test. (**D**) Myelinated axon bundle images of WT and *Sptbn4*^*geo*^ auditory brainstem (at P25). Scale bar, 0.5 µm. (**E**) (Left) Distribution of *g* ratio (inner diameter of axon divided by outer diameter of the same axon) as a function of inner axon diameter (µm). Dots indicate values from individual axons, n = 3 WT mice (in black), n = 4 *Sptbn4*^*geo*^ mice (in red). (Right) Summary of *g* ratio of WT and *Sptbn4*^*geo*^ mice, values represent means per animal ± s.e.m.

To determine whether β4-spectrin is a key molecule for structural refinement of the nerve terminal with Na_v_ channel clustering during postnatal development, we examined how loss of β4-spectrin impacts the heminode, utilizing the β4-spectrin null mice (*Sptbn4*^*geo*^). These mice were generated by gene-trap mutagenesis, where expression of the β4-spectrin protein was disrupted by ROSAβgeo* insertion^8,10^. β4-spectrin is encoded by the *Sptbn4* locus on chromosome 19q13.2^3^. In *Sptbn4*^*geo*^ mice, there was no detectable β4-spectrin expression in the MNTB located in the mouse auditory brainstem. MAP2-positive principal neurons and the calyx axon projections to the MNTB were morphologically not altered (at P25, Fig. 2A).

In WT mice, the initially broad expression of Na_v_ channels at the heminode along the calyx terminal coalesces into a cluster during the second week of postnatal age^6^. However, *Sptbn4*^*geo*^ mice did not show this structural refinement of Na_v_ channel clustering at the heminode. The calyx terminal from *Sptbn4*^*geo*^ mice displayed a significantly broader expression of Na_v_ channel along the heminode compared to the clustered Na_v_ expression observed at nodes (Fig. 2B). The Na_v_ channel cluster size at the heminode was 20.3 ± 1.9 µm in *Sptbn4*^*geo*^ mice (n = 42 cells from 6 mice) and 8.0 ± 1.0 µm in WT mice (n = 44 cells from 13 mice, *p* < 0.001, Mann–Whitney U test) without changes in the heminode length, defined as the distance between the last paranode immunostained with Caspr and the calyx terminal (12.7 ± 1.1 µm in *Sptbn4*^*geo*^ mice n = 33 from 6 mice vs 11.5 ± 1.4 µm in WT mice, n = 21 from 6 mice, P24-P34, *p* = 0.47, Mann–Whitney U test, Fig. 2C). The size of nodes, flanked by Caspr-labelled paranodes, was measured by the length of Na_v_ cluster. The loss of β4-spectrin had no effect on the length and width of nodal Na_v_ cluster in *Sptbn4*^*geo*^ mice (2.0 ± 0.14 µm, n = 38 from 3 *Sptbn4*^*geo*^ mice vs 1.7 ± 0.09 µm, n = 33 from 4 WT mice, *p* = 0.19, Mann–Whitney U test, Fig. 2C). The node length was not dependent on node diameter, determined by the width of Na_v_ cluster width. The slope of node length against node diameter was − 0.02 ± 0.27, n = 38 from in 3 *Sptbn4*^*geo*^ mice and 0.13 ± 0.16, n = 33 from 4 WT mice (*p* = 0.64, linear regression test, data not shown). Furthermore, we evaluated myelin thickness and axon diameter of axons in the MNTB, which include the calyx axons from the cochlear nucleus and MNTB axons using transmission electron microscopy (TEM). To evaluate myelin thickness, the *g*-ratio was measured as the inner radius of the axon bundle divided by the outer radius, therefore an increase in myelin thickness would be indicated by a lower *g*-ratio. There was no significant difference between the *g*-ratios of WT and *Sptbn4*^*geo*^ axon bundles (0.83 ± 0.002 g-ratio, n = 440 axon bundles in 3 WT mice, 0.83 ± 0.002 g-ratio, n = 636 axon bundles in 4 *Sptbn4*^*geo*^ mice, at P25, *p* = 0.11, Mann–Whitney U test, Fig. 2D, E) nor in the inner diameter length of the corresponding axon bundles (1.57 ± 0.10 µm in WT, 1.36 ± 0.06 µm in *Sptbn4*^*geo*^, *p* = 0.91, Mann–Whitney U test, Fig. 2D, E). These results demonstrate that the loss of β4-spectrin has no significant impact to myelin thickness or axonal diameter in the auditory brainstem. Overall, these results indicate that β4-spectrin is required for the clustering of Na_v_ channels at the heminode, but is not essential for the formation of nodes in myelinated axons in the auditory brainstem.

### β4-spectrin at the heminode is important for maintaining the reliability and temporal fidelity of presynaptic spikes at the nerve terminal

**Figure 3:**
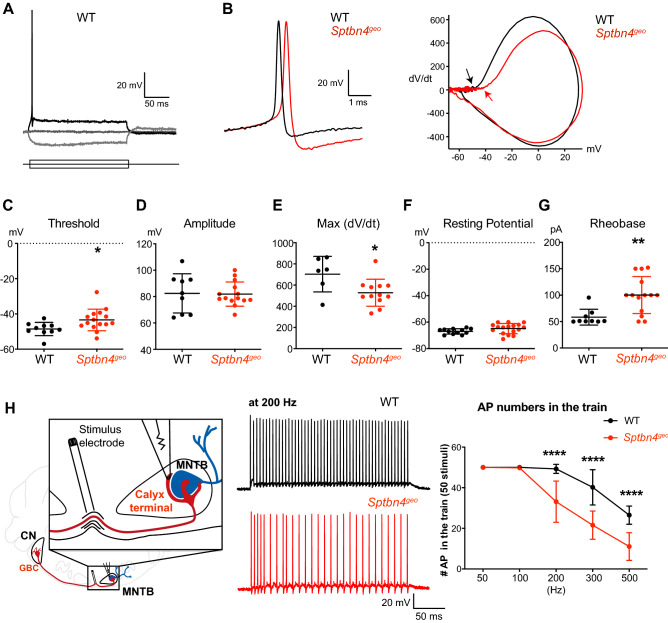
Loss of β4-spectrin reduces presynaptic excitability and increased AP failures at the nerve terminal during high-frequency stimuli. (**A**) Membrane potential changes of the calyx terminal in response to step-like current injection (− 50 to 50 pA) in whole-cell patch clamp recording in WT mouse. (**B**) Presynaptic APs elicited with a supra-threshold current injection and their corresponding phase plots (dV/dt against membrane potential) in WT (black) and *Sptbn4*^*geo*^ (red) mice. Arrows indicate the threshold of APs. (**C–G**) Summary of AP threshold and amplitude, the max dV/dt, the resting potential, and rheobase at the calyx terminal. The threshold of AP was determined by the point where dV/dt exceeds 10 V/s and the amplitude of AP from the threshold to the AP peak in the phase plot. (**H**) (Left) Diagram for presynaptic recording from the calyx of Held terminal in response to fiber stimulation placed at the midline of the brainstem. CN, cochlear nucleus,GBC, globular bushy cell, and MNTB, medial nucleus of the trapezoid body. (Middle) Representative traces of presynaptic AP train elicited with afferent axon fiber stimuli (at 200 Hz) in WT (black) and *Sptbn4*^*geo*^ (red). (Right) Summary of the number of APs in the train (50 stimuli) as a function of fiber stimuli frequencies (50 Hz to 500 Hz). values are means ± S.D. (*p < 0.05, **p < 0.01, ****p < 0.0001, Mann–Whitney U test or Two-way ANOVA).

The disruption of Na_v_ channel clustering at heminode impaired the fidelity of presynaptic action potential (AP) and increased the failure rate during a high-frequency spiking^5,17^. To determine how loss of β4-spectrin at the heminode impacts presynaptic excitability, presynaptic AP was elicited from the calyx terminal by supra-threshold current injection in whole-cell recordings (Fig. 3A, B). Our primary measures of excitability were taken from phase plots (Fig. 3B, C), plots of the membrane potential slope (dV/dt) versus the membrane potential^18^. In both WT (n = 6 mice) and *Sptbn4*^*geo*^ mice (n = 12 mice, at P14-16), all cells displayed a single inflection in the rising phase of the AP (Fig. 3C), indicating that APs generate at the heminode next to the presynaptic terminal. We used a primary criterion of the membrane potential at a phase plot slope of 10 mV ms^-1^ to estimate the threshold of presynaptic AP. The AP threshold was significantly higher in *Sptbn4*^*geo*^ mice than WT mice (− 43.3 ± 6.12 mV, n = 15 cells vs − 48.6 ± 3.79 mV, n = 10 cells, respectively, *p* = 0.012, Mann–Whitney U test) without changes in AP amplitude (81.8 ± 9.16 mV, n = 14 cells for *Sptbn4*^*geo*^ vs 82.4 ± 4.96 mV, n = 9 cells for WT, *p* = 0.816, Fig. 3B–D). In the phase plot, the maximum (Max) of dV/dt was lower in *Sptbn4*^*geo*^ mice (527 ± 127.2, n = 12 cells for *Sptbn4*^*geo*^ vs 703 ± 69.5, n = 6 cells for WT, *p* = 0.017, Mann–Whitney U test), indicating that the rise time of AP was slower in *Sptbn4*^*geo*^ mice than WT mice (Fig. 3E). The rheobase, depolarizing currents required to generate APs, was significantly larger in *Sptbn4*^*geo*^ mice compared to WT mice (100.2 ± 34.92 pA, n = 14 cells vs 58.4 ± 15.03, n = 9 cells, respectively, *p* = 0.004, Mann–Whitney U test, Fig. 3F). There was no difference in the resting potential (− 64.6 ± 3.82 mV, n = 17 cells for *Sptbn4*^*geo*^ vs − 66.9 ± 2.01 mV, n = 12 cells for WT, *p* = 0.06, Mann–Whitney U test, Fig. 3G). An elevated threshold, a slower rising of spike, and a higher rheobase at the calyx terminal indicates that lack of β4-spectrin reduced presynaptic excitability.

In addition, we examined how loss of β4-spectrin impacts the reliability and temporal fidelity of presynaptic terminal spikes during high-frequency stimulation (Fig. 3H). In WT mice, the calyx terminal was able to follow high-frequency stimulation and displayed spikes without failures at 100 Hz and 200 Hz. The calyx terminal in *Sptbn4*^*geo*^ mice showed a number of spike failures at 200 Hz (~ 30%) through 500 Hz (~ 80%). Although the failure rate was increased throughout the stimulus frequencies in both genotypes, the number of spikes in response to high-frequency stimulus were significantly decreased the calyx terminal in *Sptbn4*^*geo*^ mice (at 200 Hz, 33.1 ± 2.5 spikes/50 stimuli in *Sptbn4*^*geo*^ mice, n = 16 cells vs 49.3 ± 0.7 spikes in WT, n = 10 cells, *p* < 0.0001, two-way ANOVA). These results demonstrate that the presence of β4-spectrin at the heminode is critical for maintaining the reliability and temporal fidelity of presynaptic spikes during early postnatal development.

### Functional relevance of β4-spectrin at the nerve terminal in central auditory processing

**Figure 4:**
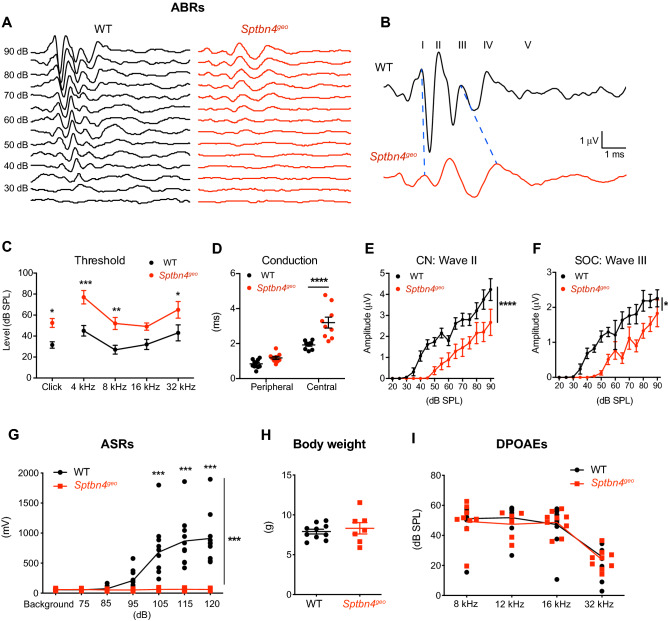
Loss of β4-spectrin impairs conduction and synchrony of neuronal activity in the central auditory brainstem. (**A**) Representative traces of ABRs in response to click stimuli (from 25 to 90 dB) from WT and *Sptbn4*^*geo*^ mice (at P25). (**B**) Magnified ABR traces in response to click stimulus at 85 dB. Roman numerals indicate waves I-V in WT mice. Blue dashed lines identify corresponding waves I and III in *Sptbn4*^*geo*^ mice. (**C**) Summary of ABR thresholds of WT and *Sptbn4*^*geo*^ mice (P21-P25) in response to click and pure tones 4, 8, 16, and 32 kHz. (**D**) Peripheral and central conduction in WT and *Sptbn4*^*geo*^ mice. Peripheral conduction was measured as the inter-peak interval between positive peaks I–II in ABRs (at 85 dB). Central conduction was measured as the time difference between positive peaks II–IV. (**E–F**) Summary of ABR amplitudes (µV) of wave II corresponding with the cochlear nucleus (CN, **E**) and wave III with the superior olivary complex (SOC, **F**) across 20 to 90 dB in WT and *Sptbn4*^*geo*^ mice. Amplitudes were measured as the µV difference between the positive peak and the preceding negative peak. ABRs: WT = 10 mice; *Sptbn4*^*geo*^ = 9 mice. (**G**) Summary of ASR maximum startle amplitudes (mV) to noise stimuli (background = 65 dB to 120 dB). (**H**) Summary of body weights (g) of WT and *Sptbn4*^*geo*^ mice. (**I**) Summary of distortion products (dB SPL) at 80 dB at various noise tones (8, 12, 16, and 32 kHz). ASRs, body weight, & DPOAEs: WT = 9 mice, *Sptbn4*^*geo*^ = 7 mice. All values plotted are means per mouse ± s.e.m. (*p < 0.05, **p < 0.01, ***p < 0.001, ****p < 0.0001 two-way ANOVA with Bonferroni’s test).

To further investigate how structural and functional alterations at the calyx axon terminal caused by β4-spectrin loss impact the auditory transmission throughout the central auditory pathway, we examined the auditory brainstem responses (ABRs) from *Sptbn4*^*geo*^ mice (P21-P25) in response to click and pure tone stimuli with varying sound pressure levels (90 to 20 dB SPL, Fig. 4A, B). There was a significant increase in ABR thresholds of *Sptbn4*^*geo*^ mice compared to WT mice when presented with click stimuli (54 ± 4.1 dB in *Sptbn4*^*geo*^ mice and 34 ± 3.4 dB in WT mice, n = 10 and 10, respectively, *p* < 0.05, two-way ANOVA with Bonferroni’s test). When presented with pure tones (4, 8, 16, and 32 kHz), the thresholds were overall much higher in the *Sptbn4*^*geo*^ mice than the WT mice (n = 10 mice for each group). The thresholds at 4 kHz (46 ± 5.1 dB in WT vs. 80 ± 4.1 dB in *Sptbn4*^*geo*^, *p* < 0.001, two-way ANOVA with Bonferroni’s test), 8 kHz (29 ± 4.0 dB in WT vs. 56 ± 4.7 dB in *Sptbn4*^*geo*^, *p* < 0.01, two-way ANOVA with Bonferroni’s test), and 32 kHz (47 ± 8.4 dB in WT vs. 70 ± 6.5 dB in *Sptbn4*^*geo*^, *p* < 0.05, two-way ANOVA with Bonferroni’s test, Fig. 4C) were significantly elevated in *Sptbn4*^*geo*^ mice when compared to WT mice, although there was no significant difference at 16 kHz (32 ± 4.4 dB in WT vs. 51 ± 8.7 dB in *Sptbn4*^*geo*^, *p* > 0.05, two-way ANOVA with Bonferroni’s test). These results indicate that *Sptbn4*^*geo*^ mice have hearing deficits.

Next, we examined peripheral and central conduction by evaluating the temporal acuity among the peaks in ABRs. Conduction was measured and determined as interpeak intervals peripherally (time between peaks I and II of ABR waves) and centrally (time between peaks II and IV). The time difference between peaks I and II of ABR waves in the WT and *Sptbn4*^*geo*^ mice were 0.84 ± 0.073 ms and 1.18 ± 0.082 ms, respectively, showing no significance difference (n = 10 and 9, respectively, *p* > 0.05, two-way ANOVA with Bonferroni’s test, Fig. 4D). These results indicate that peripheral conduction was not altered in *Sptbn4*^*geo*^ mice, which is consistent with the previous work showing a normal functioning periphery in quivering mice^19,20^. For the central conduction, *Sptbn4*^*geo*^ mice had significantly greater interpeak intervals of 3.2 ± 0.29 ms (n = 9 mice) versus 1.9 ± 0.067 ms in WT mice (n = 10 mice, *p* < 0.0001, two-way ANOVA with Bonferroni’s test). The prolonged interval indicates that central conduction was slowed down and major signal transmission mechanisms were impaired in the central auditory pathway in *Sptbn4*^*geo*^ mice. The results were comparable with those observed in patients lacking β4-spectrin^3^. In addition, we analyzed signaling strength in the auditory pathway by quantifying amplitudes of individual ABR waves as the µV difference between the positive peak and the preceding negative peak. The ABRs from *Sptbn4*^*geo*^ mice displayed significantly reduced amplitudes of waves II and III, which arise from neuronal activity in the cochlear nucleus (CN) and the superior olivary complex (SOC) (*p* < 0.0001 and *p* < 0.05, respectively, two-way ANOVA, Fig. 4E–F). Reduced ABR amplitude indicates that neuronal activity or synaptic synchrony in the CN and SOC are impaired in *Sptbn4*^*geo*^ mice. These results are consistent with the disruption of heminodal Na_v_ channel clustering and loss of the temporal fidelity of presynaptic spikes found in these mice, which causes these central hearing deficits.

To determine how the disruption in temporal fidelity of auditory impulses and transmission caused by *Sptbn4* loss influences central auditory processing, we tested the ability of mice to startle to loud noise stimuli using the acoustic startle response (ASR) test. Intriguingly, *Sptbn4*^*geo*^ mice lacking β4-spectrin were unable to elicit a startle response to any of the noise stimuli presented, whereas WT mice clearly showed a startle response with the threshold at 105 dB (*p* < 0.001, two-way ANOVA with Bonferroni’s test, Fig. 4G). Using 120 dB startle noise as the measurement for peak startle amplitude, there was a significant difference in the magnitude of the startle in *Sptbn4*^*geo*^ mice compared to WT mice (60.8 ± 5.6 mV at 120 dB, n = 7 *Sptbn4*^*geo*^ mice vs 911.3 ± 131.7 mV at 120 dB, n = 10 WT mice, *p* < 0.0001, Mann Whitney U test, Fig. 4G). To examine whether mouse body weight may influence no ASRs in *Sptbn4*^*geo*^ mice, the weights of the mice undergoing ASR testing were measured. There was no significant difference (8.3 ± 0.7 g in *Sptbn4*^*geo*^ mice, 7.9 ± 0.3 g in WT mice, *p* = 0.56, unpaired t-test, Fig. 4H). To confirm that cochlear function is normal and that cochlear deficits are not responsible for the lack of a response to startle in *Sptbn4*^*geo*^ mice, we performed the in vivo distortion product otoacoustic emissions (DPOAE) test, which measures outer hair cell function. There was no significant difference in the distortion products of pure tones presented at 8, 12, 16 or 32 kHz in *Sptbn4*^*geo*^ mice compared to WT mice (39.5 ± 8.6 dB SPL at 8 dB, 40.6 ± 7.5 dB SPL at 12 dB, 40.3 ± 6.6 dB SPL at 16 dB, 19.7 ± 2.9 dB SPL at 32 dB in *Sptbn4*^*geo*^ mice, n = 7 mice, 49.4 ± 4.5 dB SPL at 8 dB, 51.9 ± 3.5 dB SPL at 12 dB, 45.3 ± 4.6 dB SPL at 16 dB, 26.2 ± 3.3 dB SPL at 32 dB in WT mice, n = 9 mice, *p* = 0.87, two-way ANOVA, Fig. 4I), indicating that the sensory output of outer hair cell function in *Sptbn4*^*geo*^ mice is normal. Taken together, the results suggest *Sptbn4*^*geo*^ mice have relatively normal cochlear function, but have delayed and reduced central auditory transmission, which results in central auditory processing deficits.

## Discussion

We found that β4-spectrin expression and clustering at the nerve terminal occurs at a later time point compared with those at the AIS or at nodes in the auditory brainstem during postnatal development. The location of β4-spectrin is temporally associated with the structural refinement of the nerve terminal around hearing onset (P13) consistent with β4-spectrin being critical for Na_v_ channel clustering at the heminode. Although the loss of β4-spectrin severely impairs Na_v_ channel clustering at the heminode, Na_v_ channel clustering at nodes and the AIS occurs normally during postnatal development. The structural disruption of the heminode results in loss of temporal fidelity and failures of presynaptic spikes at the level of single cells and consequently reduced synchronized neuronal activities, delayed conduction in auditory brainstem circuitry, and impaired central auditory processing.

In *Sptbn4*^*geo*^ mice, loss of β4-spectrin disrupted Na_v_ channel clustering, specifically at the heminode without alterations in nodal and AIS structure, leading to elevation of AP threshold and increased failures during AP train. The results indicate that heminodal structure is critical for tuning of presynaptic spikes during postnatal development of the auditory brainstem. The correlation between the disruption of Na_v_ channel clustering at heminode and impaired temporal fidelity of presynaptic spikes was previously observed in other neuropathological models such as dys-/demyelination models^5,17^. Here, we showed that the auditory brainstem of *Sptbn4*^*geo*^ mice has normal myelination, indicating loss of temporal fidelity of auditory transmission are likely due to disruption of the heminodal structure and presynaptic failures rather than an alteration in myelination. Notably, Na_v_ channel expression at nodes and the AIS in the auditory brainstem were not altered in *Sptbn4*^*geo*^ mice (at P25), supporting the previous finding that β4-spectrin is not essential for the targeting and localization of Na_v_ channels at nodes or AIS formation^16^. However, adult mice without β4-spectrin (> 3 months old *Sptbn4*^*geo*^ mice) showed a loss of AnkG and Na_v_ channels from the AIS, and the disruption of nodal structure^8,10^. Thus, β4-spectrin is essential for maintaining membrane structure and the proper molecular organization of nodes, the AIS, and the axonal cytoskeleton^7,21^. During brain development, nodes and the AIS form properly in *Sptbn4*^*geo*^ mice, but in the adulthood or during aging, the lack of β4-spectrin results in the destabilization of the nodal and AIS structures^7,8^. Here, we found β4-spectrin is essential for Na_v_ channel clustering at the heminode rather than at nodes and AIS in the auditory brainstem during early development. The structural and functional alterations in the nerve terminal caused by loss of β4-spectrin critically impacts central auditory processing. The result suggests that the spatial and temporal expression of β4-spectrin at axon segments during development are important to determine functional relevance in different brain regions.

Central auditory processing disorders (CAPD) manifest as difficulties in certain auditory tasks such as sound localization, temporal integration, discrimination, and auditory performance with competing acoustic signals (e.g. listening in noise), without significant loss in hearing sensitivity^22^. CAPD is frequently observed in more complex systemic disorders including specific language impairment, dyslexia, attention deficits/hyperactivity disorders, and autism spectrum disorder^4^. Speech-evoked ABR onset-waves in children with CAPD had significantly longer latencies with larger variability than in their age-matched controls^23^. Alterations in temporal features of sound, which are represented by first spike latencies and their variability, impact auditory temporal processing^4^. Thus, temporal precision is a critical element for central auditory processing. However, the cellular mechanisms underlying CAPD remain unknown, due to the limited availability of animal models with CAPD.

Utilizing in vivo auditory function tests in *Sptbn4*^*geo*^ mice, we observed that there was no alteration in cochlear function with normal DPOAEs, significant impairments in ABRs, and complete loss of ASRs in *Sptbn4*^*geo*^ mice during postnatal development. Those in vivo tests evaluated the outer hair cell function, neuronal activity and conduction along the auditory processing pathway, and its sensorimotor gating ability, respectively. The results indicate that *Sptbn4*^*geo*^ mice may have impairments in sound localization and auditory processing, although they have a normal cochlear function. Notably, phenotypes in *Sptbn4*^*geo*^ mice are focused on central deficits in the auditory brainstem, although the peripheral auditory nerve deficits can be involved. We found that temporal fidelity of APs at the nerve terminal was impaired by dispersion and failures of spikes in *Sptbn4*^*geo*^ mice. The loss of temporal accuracy of APs in central processing disorders could be caused by myelin loss or deficits in myelination. For example, the structural alteration of the axon heminode and the loss of the fidelity of presynaptic spikes has been demonstrated in dys-/demyelinating axons^17^. In adult mice (> 9-month-old), loss of nodal β1- and β4-spectrins altered axon diameter and myelin thickness in the peripheral nervous system, indicating an axon injury response and motor dysfunction^13^. However, ultrastructural analysis using TEM showed that loss of β4-spectrin did not affect axonal integrity and myelin thickness in young *Sptbn4*^*geo*^ mice (~ 1 month old, Fig. 2). Thus, in juvenile mice, the loss of temporal fidelity at the nerve terminal critically contributes to central conduction and processing deficits in the auditory brainstem independent of myelin loss or axonal degeneration. The results suggest that alterations in Na_v_ channel clustering at the heminode without the disruption of nodal structure, axon integrity, and myelin can significantly affect auditory processing during development.

Humans and mice with pathogenic *Sptbn4* variants have impaired motor function, neuropathy, as well as auditory deficits^1–3^. Surprisingly, *Sptbn4*^*geo*^ mice completely lost the ability of acoustic startle response. In addition to central auditory deficits, motor dysfunctions may play a role in the inability of acoustic startle response for the *Sptbn4*^*geo*^ mice. In a previous study, the *Sptbn4*^*geo*^ mice appeared normal besides fine tremors and a progressively worsening hindlimb paralysis defined by hindlimb contraction when lifting the mice by the tail was observed beginning at 2–3 months of age^8^. However, we did not observe the clenching of the hindlimb in *Sptbn4*^*geo*^ mice at P22-P23, when ASR testing was performed (data not shown). The possibility of motor deficits playing a role in the lack of a startle reflex observed in the *Sptbn4*^*geo*^ mice is unlikely, but more likely due to auditory processing deficits. Severe motor dysfunction in *Sptbn4*^*geo*^ observed in adulthood might be caused by complex deficits including nodal disruption, myelin loss, and axonal degeneration^13^. Here we demonstrate that the lack of β4-spectrin critically impaired central auditory processing during postnatal development, prior to severe neurological phenotypes in the adult including motor dysfunction.

## Materials and methods

### Animals

*Sptbn4*^*geo*^ mice (β4-spectrin null mice), which were previously characterized in several groups^8,10^, were used. *Sptbn4*^*geo*^ mice were provided by Dr. Bhat’s laboratory (UTHSCSA). Identification was based on genotyping, with + / + individuals used as WT mice. Pups of either sex were used for presynaptic recordings (at P14–16) and for in vivo ABR, ASR, and DPOAEs test and immunostaining (P22–P34). Experiments were in accordance with animal welfare laws, complied with ARRIVE guidelines and protocols approved by the University of Texas Health Science Center San Antonio (UTHSCSA) Institutional Animal Care and Use Committee (#20140045AR).

### In vivo auditory brainstem response (ABR) test

ABR recordings were performed as described (Kim et al., 2013). Briefly, mice were anesthetized with 3.5% isoflurane and maintained with 2.5% isoflurane during recording (1 l/min O_2_ flow rate). ABR recordings were performed in a sound attenuation chamber (Med Associates, Albans, VT). Subdermal needle electrodes (Rochester Electro-Medical, Lutz, FL) were placed on the top of the head, ipsilateral mastoid, and contralateral mastoid as the active, reference, and ground electrode, respectively. The signal differences in the ABRs between the vertex and the mastoid electrodes were amplified and filtered (100–5000 Hz). Acoustic stimuli were generated by an Auditory Evoked Potentials Workstation (Tucker-Davis Technologies [TDT], Alachua, FL). Closed-field click stimuli were presented to the left ear. The signals consisted of a series of amplitude-modulated square waves (0.1 ms duration, 16/s) through TDT Multi-Field Magnetic Speakers. The sound stimuli were delivered through a 10-cm plastic tube (Tygon; 3.2-mm outer diameter) at a repeat rate of 16/s. Sound intensities ranged from 90 to 20 dB, with 5-dB decrements, and responses to 512 sweeps were averaged.

### Acoustic startle response test

Mice of either sex between ages P22-P23 were put in a Plexiglas holding cylinder located in sound-attenuated chamber using the SR-LAB startle response system (San Diego Instruments, San Diego, CA). Weights of mice were recorded to account for sex or size differences. Sound levels from the chamber speakers were calibrated with a digital sound level meter (Part Number MS-M80A, Mengshen). Each trial consisted of an initial acclimation period of 5 min of background level noise (at 65 dB) followed by 5 rounds of randomized noise stimuli playing for 20 ms with 10 s of background between each noise stimuli at 65 dB (background), 95 dB, 105 dB, 115 dB, and 120 dB. Stimuli was produced by a digital signal processing-controlled system amplified and emitted by a loudspeaker. Startle responses were measured inside the sound-attenuated chamber by a movement-sensitive piezo-accelerometer platform. The maximum was used as the startle amplitude at each tone with millivolts (mV) as the unit of measurement and averaged per mouse.

### Distortion product otoacoustic emissions (DPOAE) test

Mice were anesthetized with 3.5% isoflurane and maintained with 2.5% isoflurane during recording. DPOAE recordings were performed in a sound attenuation chamber (Med Associates, Albans, VT). Acoustic stimuli were generated by an Auditory Evoked Potentials Workstation (Tucker-Davis Technologies [TDT], Alachua, FLO). The ER-10B + recording microphone (Etymotic Research, Elk Grove Village, IL) with ear-tip was inserted into the ear canal. The sound stimuli were delivered through two TDT Multi-Field Magnetic Speakers connected to the recording microphone by 10-cm coupling tubes (TDT; 3.2 mm outer diameter). Pure tones were presented at 20% frequency separation between f1 and f2 at 8, 12, 16, and 32 kHz. Sound intensities ranged from 80 to 20 dB, with 10-dB decrements, and responses to 512 sweeps were averaged. Distortion products were calculated as 2f1-f2 minus the noise floor that were detected by the recording microphone and amplified by RZ6 processor (TDT).

### Slice preparation

After rapid decapitation of the mice, the brainstem was quickly removed from the skull and immersed in ice-cold low-calcium artificial CSF (aCSF) containing the following (in mM): 125 NaCl, 2.5 KCl, 3 MgCl_2_, 0.1 CaCl_2_, 25 glucose, 25 NaHCO_3_, 1.25 NaH_2_PO_4_, 0.4 ascorbic acid, 3 myoinositol, and 2 Na-pyruvate, pH 7.3–7.4 when bubbled with carbogen (95% O_2_/5% CO_2_), and 310–320 mOsmol/L. The brainstem was sectioned (200 μm thick for electrophysiological recordings and immunostaining) and the slices were transferred to an incubation chamber containing normal aCSF bubbled with carbogen, where they were maintained for 30 min at 35 °C and thereafter at room temperature (24 °C). Normal aCSF was the same as low-calcium (slicing) aCSF, but with 1 mM MgCl_2_ and 2 mM CaCl_2_.

### Electrophysiology

Slices were perfused with normal aCSF at 2 ml/min and visualized using an infrared differential interference contrast microscope (AxoExaminer, Zeiss, Oberkochen Germany) with a 63 × water-immersion objective and a CMOS camera (ORCA-Flash2.8, Hamamatsu, Japan). Whole-cell patch-clamp recordings were performed in normal aCSF at room temperature (24 °C) using an EPC-10 amplifier controlled by PATCHMASTER software (HEKA, Elektronik, Lambrecht/Pfalz, Germany). For presynaptic recordings, the pipette solution contained (in mM): 125 K-gluconate, 20 KCl, 5 Na_2_-phosphocreatine, 10 HEPES, 4 Mg-ATP, 0.2 EGTA, and 0.3 GTP, pH adjusted to 7.3 with KOH. Recordings were not corrected for the predicted liquid junction potential of 11 mV. Patch electrodes had resistances of 4–5 MΩ. Current-clamp recordings were continued only if the initial uncompensated series resistance was < 20 MΩ^5,24^. Lucifer Yellow (1 mM, Invitrogen) was added to the pipette solution to visualize the calyx of Held terminal. Presynaptic APs from the calyx of Held terminal were evoked by stimulation with a bipolar platinum-iridium electrode (Frederick Haer, Bowdoinham, ME) placed near the midline spanning the afferent fiber tract of the MNTB. An Iso-Flex stimulator driven by a Master 10 pulse generator (A.M.P.I., Jerusalem, Israel) delivered 100-µs pulses at 1.2 times threshold (< 15 V constant voltage). The average threshold of stimulation intensity was 1.4 ± 0.23 V. Signals were filtered at 2.9 kHz and acquired at a sampling rate of 10–50 µs. Data were analyzed offline and presented using Igor Pro (Wavemetrics, Lake Oswego, OR). Presynaptic AP trains were obtained by averaging three sweeps (five for a single AP) in each experiment.

### Immunohistochemistry

For anterograde tracer labeling, 1 mg of tetramethylrhodamine-dextran (Invitrogen) was injected to the midline of the acute brainstem slices (200 µm) using a 21Gx 1½ (0.8 mm × 40 mm) needle, which were then incubated in normal aCSF bubbled with carbogen at 37 °C for 30 min. All slices were fixed with 4% (w/v) paraformaldehyde in PBS for 10 min. Free-floating sections were blocked in 3% goat serum and 0.3% Triton X-100 in PBS for 30 min. Slices were incubated with the primary antibody overnight at room temperature. The following primary antibodies were used: mouse anti-sodium channel (PanNa; 1:400; Sigma), rabbit anti-calretinin (CR; 1:200; Invitrogen), guinea pig anti-Caspr (Caspr; 1:200; from Dr. Bhat’s laboratory, UTHSCSA), rabbit anti-β4-spectrin (β4; 1:200; from Dr. Bhat’s laboratory, UTHSCSA), mouse anti-ankyrinG (AnkG; 1:200; Neuromab). Antibody labeling was visualized by incubation with appropriate Alexa dye–conjugated secondary antibodies (1:500; Invitrogen) for 2 h at room temperature. Stained slices were viewed with laser lines at 488 nm, 568 nm, and 647 nm using a 40x/1.40 or 63 × /1.40 oil-immersion objective on a confocal laser-scanning microscope (LSM-710; Zeiss). Stack images were acquired at a digital size of 1024 × 1024 pixels with optical section separation (*z*-interval) of 0.5 µm and were later crop to the relevant part of the field without changing the resolution. The heminode length was measured from the point at the outer edge of the principal neuron at the neck of the calyx terminal to the beginning of detectable Caspr at the heminode. The Na_v_ channel cluster size at the heminode was measured as the length from beginning to end of the Na_v_ cluster expression, and the paranodal length and width was measured from Na_v_ channel expression mentioned previously^6^. The confocal image stacks were analyzed using ImageJ software.

### Transmission electron microscopy

The brain was removed and immersed in ice-cold low-calcium aCSF (mentioned previously in slice preparation) and the MNTB from the brainstem was dissected at 200 µm-thick section using a microtome (VT1200s, Leica), then fixed in a 4% Formaldehyde—1% Glutaraldehyde solution and stored at 4 °C. Further processing was performed by the UTHSCSA Electron Microscopy Lab as previously described^25,26^. Axon bundle images were analyzed at a final magnification of 8000X, with the longest length of the axon measured as the inner diameter and the inner radius divided by the outer radius as the *g*-ratio. Three images of axon bundles per mouse were analyzed and averaged.

### Statistical analysis

Experimental data were analyzed and presented using Igor Pro and Prism (GraphPad Software, San Diego, CA). For statistical significance, we tested the normality of the data distribution with the Kolmogorov–Smirnov test with the Dallal-Wilkinson-Lillie correction for corrected *p *values using Prism 5. If a dataset passed the normality test, we used the unpaired Student’s *t*-test; for all other datasets we used the non-parametric Mann-Whiney U test. For statistical significance of multiple groups, we used a two-way ANOVA. For comparing slopes of lines, we used the linear regression test. Data collected as raw values are shown as mean ± S.E.M. or mean ± S.D. Details of statistical methods are reported in the text. For all analyses, *p *values < 0.05 were considered significant.

## Supplementary Information

Supplementary Information.

## Data Availability

All data generated or analyzed during this study are included in this published article (and its Supplementary Information files).

## References

[1] Wang C-C (2018). βIV spectrinopathies cause profound intellectual disability, congenital hypotonia, and motor axonal neuropathy. Am. J. Human Genet..

[2] Parkinson NJ (2001). Mutant beta-spectrin 4 causes auditory and motor neuropathies in quivering mice. Nat. Genet..

[3] Knierim E (2017). A recessive mutation in beta-IV-spectrin (SPTBN4) associates with congenital myopathy, neuropathy, and central deafness. Hum. Genet..

[4] Kopp-Scheinpflug C, Tempel BL (2015). Decreased temporal precision of neuronal signaling as a candidate mechanism of auditory processing disorder. Hear. Res..

[5] Berret E, Kim SE, Lee SY, Kushmerick C, Kim JH (2016). Functional and structural properties of ion channels at the nerve terminal depends on compact myelin. J. Physiol..

[6] Xu J, Berret E, Kim JH (2017). Activity-dependent formation and location of voltage-gated sodium channel clusters at a CNS nerve terminal during postnatal development. J. Neurophysiol..

[7] Yang Y (2004). Spectrins are essential for membrane stability and the molecular organization of nodes of Ranvier. J. Neurosci..

[8] Komada M, Soriano P (2002). βIV-spectrin regulates sodium channel clustering through ankyrin-G at axon initial segments and nodes of Ranvier. J. Cell Biol..

[9] Liu, C.-H. & Rasband, M.N. Axonal Spectrins: Nanoscale Organization, Functional Domains and Spectrinopathies. *Front. Cell. Neurosci.* **13**, 234. 10.3389/fncel.2019.00234 (2019). 10.3389/fncel.2019.00234 PMC6546920 31191255

[10] Saifetiarova J, Shi Q, Paukert M, Komada M, Bhat MA (2018). Reorganization of Destabilized Nodes of Ranvier in βIV SpectrinMutants Uncovers Critical Timelines for Nodal Restoration and Prevention of Motor Paresis. J. Neurosci..

[11] Yang Y, Ogawa Y, Hedstrom KL, Rasband MN (2007). βIV spectrin is recruited to axon initial segments and nodes of Ranvier by ankyrinG. J. Cell Biol..

[12] Liu, C.-H. *et al.* β spectrin-dependent and domain specific mechanisms for Na+ channel clustering. *eLife* **9**, e52378. 10.7554/eLife.52378 (2020). 10.7554/eLife.56629 PMC7237202 32425157

[13] Liu, C.-H. *et al.* Nodal β spectrins are required to maintain Na+ channel clustering and axon integrity. *eLife* **9** (2020). 10.7554/eLife.52378 PMC7018506 32052742

[14] Ho TS (2014). A hierarchy of ankyrin-spectrin complexes clusters sodium channels at nodes of Ranvier. Nat. Neurosci..

[15] Ford MC (2015). Tuning of Ranvier node and internode properties in myelinated axons to adjust action potential timing. Nat. Commun..

[16] Yang Y, Ogawa Y, Hedstrom KL, Rasband MN (2007). betaIV spectrin is recruited to axon initial segments and nodes of Ranvier by ankyrinG. J. Cell Biol..

[17] Kim JH, Kushmerick C, von Gersdorff H (2010). Presynaptic resurgent Na+ currents sculpt the action potential waveform and increase firing reliability at a CNS nerve terminal. J. Neurosci..

[18] Bean BP (2007). The action potential in mammalian central neurons. Nat. Rev. Neurosci..

[19] Bock GR, Frank MP, Steel KP, Deol MS (1983). The quivering mutant mouse: hereditary deafness of central origin. Acta Otolaryngol..

[20] Deol MS, Frank MP, Steel KP, Bock GR (1983). Genetic deafness of central origin. Brain Res..

[21] Lacas-Gervais S (2004). BetaIVSigma1 spectrin stabilizes the nodes of Ranvier and axon initial segments. J. Cell Biol..

[22] Stefanatos GA, Demarco AT (2012). Central auditory processing disorders.

[23] Rocha-Muniz CN, Befi-Lopes DM, Schochat E (2014). Sensitivity, specificity and efficiency of speech-evoked ABR. Hear. Res..

[24] Kim SE, Turkington K, Kushmerick C, Kim JH (2013). Central dysmyelination reduces the temporal fidelity of synaptic transmission and the reliability of postsynaptic firing during high-frequency stimulation. J. Neurophysiol..

[25] Barron T, Saifetiarova J, Bhat MA, Kim JH (2018). Myelination of Purkinje axons is critical for resilient synaptic transmission in the deep cerebellar nucleus. Sci. Rep..

[26] Kim EJ, Nip K, Blanco C, Kim JH (2021). Structural refinement of the auditory brainstem neurons in Baboons during perinatal development. Front. Cell Neurosci..

