# Supplementary material for: Loss of β4-spectrin impairs Nav channel clustering at the heminode and temporal fidelity of presynaptic spikes in developing auditory brain
Source: Sci Rep. 2022 Apr 7;12:5854. doi: 10.1038/s41598-022-09856-9 (PMC8991253; doi:10.1038/s41598-022-09856-9)
Supplement: Supplementary file 1 — Supplementary Information. [file 41598_2022_9856_MOESM1_ESM.pdf]

Supplemental Figure 1

P25

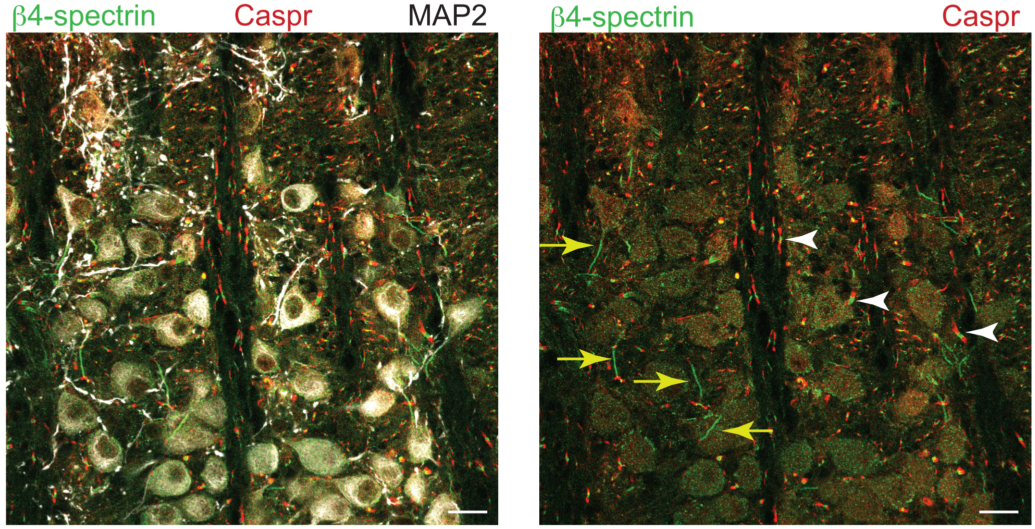

P9

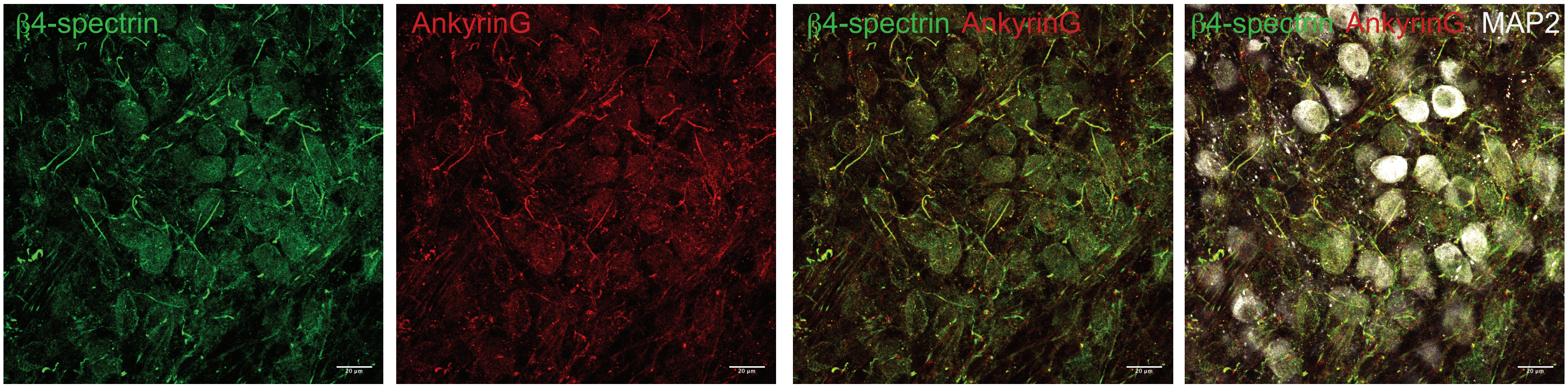

Supplemental Figure 1. β4-spectrin is expressed at the node of Ranvier (nodes) and the axon ininital segments (AIS). (Top) The MNTB was immunolabeled with β4-spectrin, Caspr, and MAP2. At P25, β4-spectrin is well located at nodes (white arrows) and the AIS (yellow arrows). (Bottom) At P9, β4-spectrin (green) is well co-localized with AnkyrinG (red) at nodes and the AIS.
